# Mechanobiological insight into brain diseases based on mechanosensitive channels: Common mechanisms and clinical potential

**DOI:** 10.1111/cns.14809

**Published:** 2024-06-25

**Authors:** Bolong Li, An‐ran Zhao, Tian Tian, Xin Yang

**Affiliations:** ^1^ Shenzhen Key Laboratory of Translational Research for Brain Diseases, the Brain Cognition and Brain Disease Institute, Shenzhen Institute of Advanced Technology Chinese Academy of Sciences Shenzhen Guangdong China; ^2^ College of Life Sciences University of Chinese Academy of Science Beijing China; ^3^ Faculty of Life and Health Sciences Shenzhen University of Advanced Technology Shenzhen Guangdong China

**Keywords:** Ca^2+^ signal, low‐intensity ultrasound stimulation, mechanosensitive channels, neurodegenerative diseases, Piezo1, place‐occupying damage

## Abstract

**Background:**

As physical signals, mechanical cues regulate the neural cells in the brain. The mechanosensitive channels (MSCs) perceive the mechanical cues and transduce them by permeating specific ions or molecules across the plasma membrane, and finally trigger a series of intracellular bioelectrical and biochemical signals. Emerging evidence supports that wide‐distributed, high‐expressed MSCs like Piezo1 play important roles in several neurophysiological processes and neurological disorders.

**Aim**s**:**

To systematically conclude the functions of MSCs in the brain and provide a novel mechanobiological perspective for brain diseases.

**Method:**

We summarized the mechanical cues and MSCs detected in the brain and the research progress on the functional roles of MSCs in physiological conditions. We then concluded the pathological activation and downstream pathways triggered by MSCs in two categories of brain diseases, neurodegenerative diseases and place‐occupying damages. Finally, we outlined the methods for manipulating MSCs and discussed their medical potential with some crucial outstanding issues.

**Results:**

The MSCs present underlying common mechanisms in different brain diseases by acting as the “transportation hubs” to transduce the distinct signal patterns: the upstream mechanical cues and the downstream intracellular pathways. Manipulating the MSCs is feasible to alter the complicated downstream processes, providing them promising targets for clinical treatment.

**Conclusions:**

Recent research on MSCs provides a novel insight into brain diseases. The common mechanisms mediated by MSCs inspire a wide range of therapeutic potentials targeted on MSCs in different brain diseases.

## Graphical abstract

The mechanosensitive channels transduce the extracellular mechanical signal (stiffness, pressure, and compression, etc.) to intracellular downstream biochemical signals, which serve as common mechanisms for pathophysiological processes in the brain, and provide potential non‐invasive targets for ultrasound stimulation.

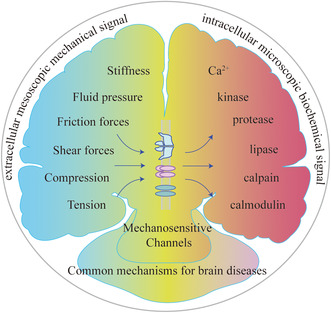

## 1 INTRODUCTION

The tiny mechanical cues carry indispensable information, especially in the softest organ, the brain.^1^, ^2^ The cell‐matrix interaction, cell–cell interaction, and liquid flow generate mechanical cues in the brain, including compression, tension, friction, shear forces, and stiffness.^3^ Transmitting through the extracellular matrix, plasma membrane, and cytoskeleton, the mechanical cues are finally sensed by the neural cells. The mechanical cues activate the mechanosensitive channels (MSCs) broadly expressed in neural cells and trigger a permeation of ions or small molecules through the membrane. There are many MSCs expressed in the brain, including Piezo1 and Piezo2, and numbers of transient receptor potential (TRP) channel super‐families.^3^, ^4^, ^5^, ^6^ The MSCs convert the mechanical stimulation into intracellular downstream biochemical and bioelectrical signals, leading to changes in the metabolism and behaviors of the local neural cells. Therefore, understanding the functional roles of MSCs provides a novel mechanobiological perspective for neurological processes.

Throughout the lifespan, the MSCs regulate neurological processes. Emerged recent research interrogated how a specific MSC acts as an upstream trigger of a neurophysiological process, such as neurogenesis, cerebral blood vessel developing, and neurovascular coupling, in which the local mechanical cues are sensed by MSCs. Very similar conditions also occur in a variety of brain diseases including neurodegenerative diseases, stroke, brain tumors, etc., in which the mechanical cues changed markedly. For example, the misfolded protein deposits in neurodegenerative diseases are much stiffer than brain tissue, which upregulates the expression and activation of Piezo1. In another condition, the edema caused by ischemic stroke generates larger compression among the adjacent cells, which activates Swell and permeates Cl^−^ and amino acids. Overall, the hyperactivation and dysregulation of MSCs triggers pathological processes in brain diseases, but the lack of a forethoughtful knowledge system for the underlying common mechanisms has stalled the basic and translational research focused on MSCs.

As MSCs are emerging potential targets for several brain diseases, an expanding corpus of studies posit that manipulating MSCs via low‐intensity ultrasound stimulation (LIUS) or modulators for MSCs would be promising therapeutic strategies for brain diseases. Though clinical trials showed that ultrasound neuromodulation is safe for human subjects, the combined mechanisms of LIUS mediated by MSCs or the other targets is largely unknown, which prevents the widespread use of LIUS for treating brain diseases clinically.

Here, for the first time, we illustrate the underlying common mechanisms of multiple different neurophysiological processes and neurological diseases from a mechanobiology perspective focused on MSCs. We first describe the mechanical cues and MSCs detected in mammalian brain, and their crucial roles in cerebral neurophysiological processes. Next, through summarizing the molecular mechanisms related to MSCs in neurodegenerative diseases and place‐occupy damage, we highlight the relatively simple MSCs act as “transport hubs” to bridge the gap between various mesoscopic mechanical cues and the extremely sophisticated downstream intracellular pathways, providing promising therapeutic targets for several brain diseases. In addition, we briefly conclude the MSCs‐mediated mechanisms and clinical trials of LIUS and draw a blueprint for the clinical manipulation of MSCs to treat brain diseases, as well as the potential side effects and outstanding issues, which we believe will inspire the following researchers.

## 2 AN OVERVIEW OF MECHANICAL CUES AND MSCs

### 2.1 Mechanical cues in the brain

Mechanical forces in the brain are generated from brain parenchyma and biofluid. The most widely used parameter to describe mechanical cues of the brain parenchyma is stiffness, which represents the potential for deformation. The brain stiffness can be quantified robustly via atomic force microscope (AFM) in brain slices and cultured cells, or via magnetic resonance elastography (MRE) in living humans and animals, and is measured quantitatively in terms of Young's modulus (Pa).^3^ A larger Young's modulus usually represents the tissue is stiffer. The stiffness of brain parenchyma supplies strain forces that inflict on the adjacent matrix or cells, which would cause deformation‐related membrane and cytoskeleton tension. The brain tissue has been observed to turn stiffer with brain development and when neurons are activated; while turned softer when aging or in the process of neurodegenerative diseases.^7^

**FIGURE 1:**
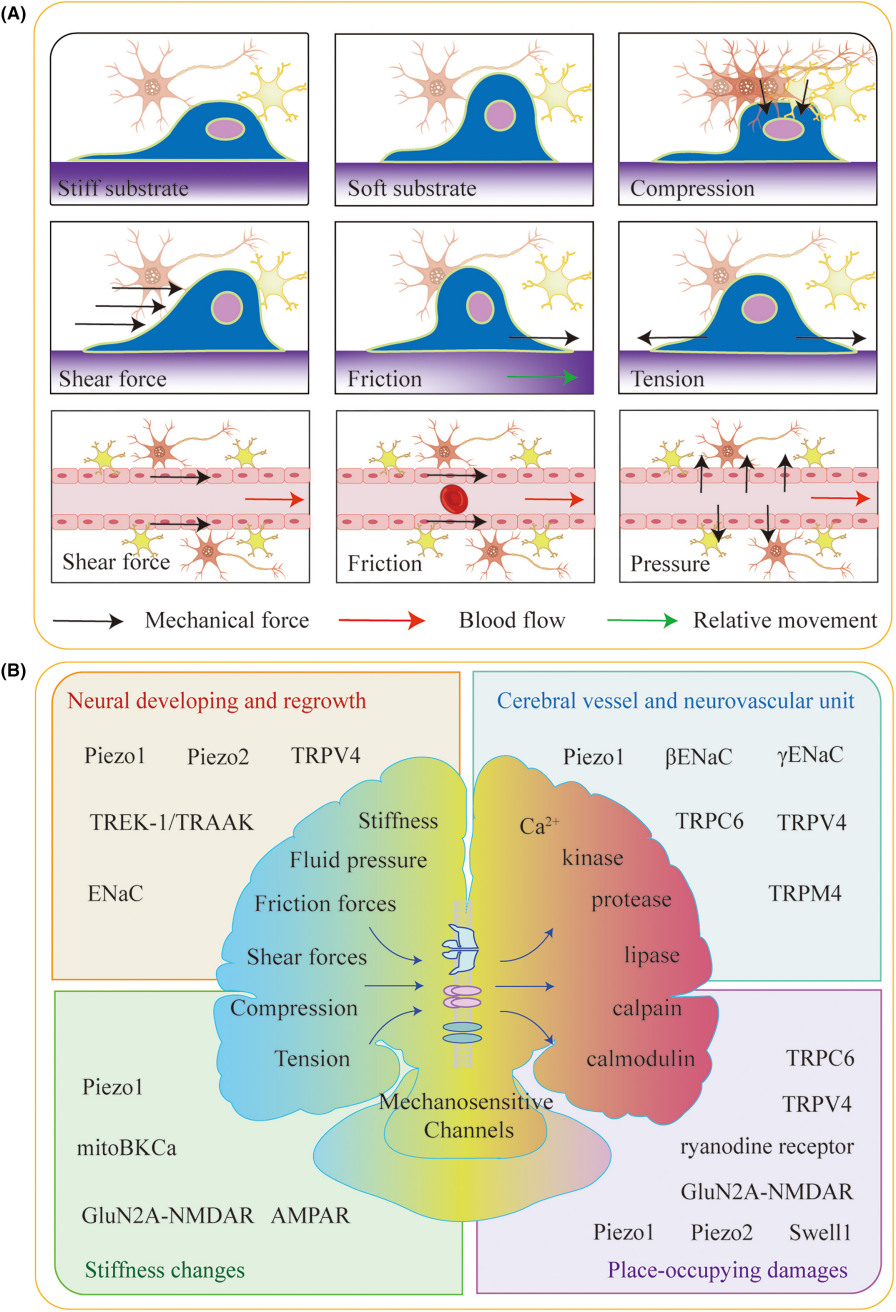
The schematic diagram for the mechanical cues and functions of MSCs in the brain. (A) The forces, including compression, shear force, friction, tension, and fluid pressure, are shown as the black arrows and the deformations of the cells. The effect of substrate stiffness is also displaced. The mechanical conditions in the niche are composed of these mechanical cues concluded here. (B) The members of MSCs participate in the neurophysiological and neuropathological processes are shown, including neural developing and regrowth (upper left), cerebral vessel and neurovascular unit (upper right), neurodegenerative diseases with changed stiffness (bottom left), and place‐occupying damages occurred with brain injury including stroke, TBI and brain tumor (bottom right). In the middle, we show the signal patterns, the extracellular mesoscopic mechanical signal (left), and the intracellular microscopic biochemical signal (right).

The parenchyma mechanical forces include the following types, the compression, tension, and friction (Figure 1).^8^, ^9^, ^10^ Besides endogenous mechanical cues, the forces generated from outside the brain (i.e., traumatic brain injury, TBI) can also affect the neural cells. The extreme exogenous mechanical forces might even break the neurites.^11^ There are mechanical forces associated with cerebral vessels and ventricles, and the fluid accommodated in the lumen. The cerebral fluid‐associated mechanical forces are shear forces and biofluid pressure. Friction is also generated and applied to capillary endothelium due to the similar size between capillary inner diameter and blood red cells.^12^ The amplitude of arteriole dilatation is partly dependent on the stiffness or elasticity of the local tissue. The cortex‐surface arterioles immersed in cerebrospinal fluid can dilate drastically compared with those embedded in the brain parenchyma, indicating that the cortex‐surface arterioles might impose a smaller pressure when dilatation than the parenchyma‐embedded arterioles.^7^

### 2.2 Mechanosensitive channels in the brain

When a mechanical force acts on the MSCs, the channels can be opened. Thus, the channel‐specific ions such as Ca^2+^, Na^+^, K^+^, and Cl^−^ would be permeated through the opened MSCs. There are MSCs permeate amino acids accompanied by ions as well. For example, the volume‐regulated anion channels (VRACs) that comprise Swell1 (also known as LRRC8A, leucine‐rich repeat‐containing protein 8a) permeates small molecules like glutamate, aspartate, and taurine under pathological activation.^13^, ^14^ The MSC‐associated flux of ions and molecules results in converting the mechanical information into intracellular signaling. The MSCs expressed in the brain are listed in Table 1.

**TABLE 1:** The MSCs expressed in the brain.

Super‐family	Member	Permeability	Main localization in mammalian brain	References
Piezo	Piezo1	Ca^2+^, Na^+^, K^+^	Broadly expressed in whole brain level and cell types, including neural stem cells, neurons, astrocytes, microglia, oligodendrocyte, endothelial tip cells, and glioma cells	15, 16, 17, 18, 19
	Piezo2	Ca^2+^, Na^+^, K^+^	Medulloblastoma cells	^20^
TRP	TRPC1	Ca^2+^	Broadly expressed in whole brain level. In Soma, Dendrites, and Axons of Hippocampal neuron and neocortical somatostatin interneurons. In brain microvessel endothelial cells	21, 22
	TRPC5	Ca^2+^	Broadly expressed in whole brain level, especially in pyramidal cells	^23^
	TRPC6	Ca^2+^	In the smooth muscle cells of middle cerebral artery, and glioma cells	24, 25, 26
	TRPV4	Ca^2+^	In astrocyte, especially at astrocytic endfeet warpping the cerebral vessel	^27^
	TRPA1	Ca^2+^	In astrocyte	^28^
	TRPP2	Ca^2+^	At brain microvessel endothelial cells. At ER membrane of cerebral vascular smooth muscle	^21^
ENaC	ENaC/ASIC1a	Na^+^	Whole brain, neural stem cells and neuroblasts in Subependymal zone and hippocampus subgranular zone, and neurons	^29^
K2P	TRAAK	K^+^	Nodes of Ranvier of myelinated neurons	30, 31
	TREK‐1	K^+^	Nodes of Ranvier of myelinated neurons	^30^
VRAC	Swell1	Cl^−^ and some amino acids	In neuron, astrocyte, and microglia	13, 32, 33
Glu receptors	GluN2B‐NMDAR	Ca^2+^, Na^+^	Broadly expressed in whole brain level	^5^
	AMPAR	Ca^2+^, Na^+^	Broadly expressed in whole brain level	^34^
mitoBKCa	mitoBKCa	K^+^	In mitochondria	^35^
Ryanodine receptor	Ryanodine receptor	Ca^2+^	In endoplasmic reticulum	36, 37

*Note*: The localization of the MSCs is generally summarized from those studies interrogated the mechanotransduction properties of MSCs, thus ensure the distribution of MSCs listed here are not mostly gated by the non‐mechanical cues in neurophysiological or neuropathological processes. Because many of the MSCs, especially TRP family members, could be also activated by other factors.

Generally, the molecular mechanisms of MSCs can be described by two models, one is the force‐from‐lipids model and the other is the force‐from‐filaments model.^3^ Force‐from‐filaments model suggests that when a mechanical force perturbates the tether proteins that the MSCs anchoring with, usually the cytoskeleton (i.e., microtubules and actin filaments) and/or the extracellular matrix protein (i.e., collagen IV and N‐linked glycans), they pull the subunits of the MSCs and change their conformation, thus opening the channels. The opening of the Piezo2, epithelial sodium channel (ENaC), TRPA1 and TRPV4 follow the force‐from‐lipids model.^38^ Force‐from‐lipids model suggests that when force perturbates on membrane lipid bilayer, the lipid organizations and composition are altered, inducing conformational changes and opening of the MSCs. The opening of the Piezo1 and numbers of two‐pore domain potassium (K2P) channels including TREK‐1 and TARRK, follow the force‐from‐lipids model.^39^

It should be emphasized that some MSCs are usually famous as ligand‐gated channels; while they could also be opened in pathological mechanical conditions. For example, the AMPAR and GluN2B‐containing NMDAR permeate Ca^2+^ when binding with glutamate.^5^ The TRP channels could be gated by other factors like temperature and ligands as well. In the past decades, however, whether the functional roles of these MSCs are mediated by the mechanotransduction properties has not been discussed in a stringent criterion. Moreover, evidence supports that Piezo1 would regulate neural cell behaviors without channel activity, but whether the MSCs are coupled with intracellular ligands and interaction proteins (i.e., Piezo1 interacts with the sarcoplasmic/endoplasmic reticulum Ca^2+^ ATPase 2 (SERCA2)) are largely unknown.^40^

In the future, there might be novel MSCs to be identified, and there might also be some known channels whose mechanotransduction properties are discovered and verified.

## 3 FUNCTIONAL ROLES OF MSCs IN THE CEREBRAL NEUROPHYSIOLOGICAL PROCESSES

### 3.1 Piezo and K2P channels regulate neural developing

Throughout the whole lifespan, the mechanical cues are changed in the brain. The brain is generally stiffened with brain development, and softened with aging.^41^, ^42^, ^43^, ^44^ In the embryonic mouse brain, the radical glial cells and neurons are major contributors to the increased stiffness with development, while the neural progenitors contribute little to stiffness.^45^ The stabilization and maturation of neural microtubule cytoskeleton and an unequal intracellular distribution of organelles contribute to the stiffening.^2^, ^45^ Besides, some other mechanical cues might also be triggers for brain development and enlargement, such as an increased cerebrospinal fluid pressure.^46^, ^47^

The changed mechanical cues act as both the consequence of brain development and an unclear trigger of an ongoing brain developing stage.^48^ The cell lineages differentiated from neural progenitor/stem cells are partly dependent on the matrix stiffness. The neuronal differentiation and neurite elongation are favored on the softest substrate where Young's modulus is among 1 kPa, instead of on the surface of those stiff matrix.^49^, ^50^, ^51^ The other glial lineage differentiation is favored on the stiffer matrix.^49^ The Piezo family and other MSCs sense the mechanical cues that change in different developing stages, thus altering the behavior of neural stem cells. The deficiency of Piezo1 in neural stem cells results in astrogenesis but not neurogenesis^15^; while the activation of Piezo1 enhances neurogenesis via triggering the downstream BMP2/Smad pathway.^52^

After regulating differentiation, Piezo1 then participates in the wiring of neural circuits and networks. For axonal growth and pathfinding, Piezo1 senses the stiffness gradient and guides the axon growth toward the softer tissue.^53^ Piezo1 is also necessary for the dendrite targeting of the projection neuron though the Ca^2+^ permeability of Piezo1 is dispensable, indicating that some Piezo1‐coupled downstream pathways are triggered without Ca^2+^ transients.^54^ However, the downstream details of the Piezo1 are still unclarified.

The process of myelination stiffens the brain in the following developing progress; while Piezo1 serves as an inhibitor for excessive myelination.^55^, ^56^ Suppression of Piezo1 in CNS, PNS, and intracerebral hemorrhage would alleviate demyelination.^57^, ^58^, ^59^ However, in Schwann cells of the PNS, activating Piezo2 is observed to promote myelination,^58^ indicating different downstream mechanisms of the Piezo1 and Piezo2, because the diversified downstream pathways might not been triggered only by a Piezo‐associated Ca^2+^ influx. These phenomena suggest Piezo1 and Piezo2 would be promising targets for treating demyelination‐associated diseases, such as AD, MS, and TBI. Besides, the K2P channels are expressed at the node of Ranvier between two bands of axonal myeline. The TREK‐1 and TRAAK could be activated by membrane stretch transducing along the axon, which subsequently suppresses the neuronal excitability.^60^

### 3.2 Piezo1 and ENaC regulate adult neurogenesis

In adult mice, neurogenesis and regrowth are associated with mechanical cues and MSCs. The neural stem cells in the murine subependymal zone express ENaC, which is activated by the fluid flow and causes an influx of Na^+^, followed by the opening of endoplasmic reticulum (ER) store‐operated Ca^2+^ channels. The enhanced Ca^2+^ elevates the level of phosphorylated Erk kinase and subsequently leads to adult neurogenesis.^29^ The deficiency of astrocytic Piezo1 in the hippocampus leads to a decrease in neurogenesis via an ATP‐dependent downstream pathway, resulting in cognitive impairment.^16^

However, the mechanical stimulation activates neuronal Piezo1 causing an inhibition of axonal regeneration in adults. The activated neuronal Piezo1 triggers the synthesis of nitric oxide (NO) and then restricts axonal growth via upregulating two kinase families. One is cyclic guanosine monophosphate (cGMP) kinases including Foraging and PKG^17^; another is Atr, a serine/threonine kinase that can detect DNA damage by phosphorylating Chek1, followed by inhibiting Cdc25 and shutting down axonal regrowth.^61^ This phenomenon indicates that the downstream pathways of neuronal and astrocytic Piezo1 is distinctly different, thus it should be discussed carefully on the potential side effects caused by the other cell types when utilizing a regulator of MSCs to manipulate them.

### 3.3 Piezo1 regulates cerebral blood vessels developing

In the processes of brain vascular development, the blood flow activates vascular endothelial Piezo1 and upregulates the Notch signaling, followed by an increasing level of Notch ligands in vascular endothelial cells, and finally activates the Notch signaling in the adjacent pericytes, which enhances the proliferation of pericytes.^62^ The scabbarded pericytes on the cerebral vessels are crucial parts of the blood–brain barrier and neurovascular units.

Besides, the Piezo1‐dependent NO‐associated pathway also plays an important role in cerebral vascular pathfinding, which is regulated by those expressed in endothelial tip cells. In the vascular branch that is toward the stiffer condition, high‐frequency Ca^2+^ influx leads to an increased calpain expression, which then disassembles the focal adhesion proteins anchored on the membrane to disintegrate the cytoskeleton, preventing vascular growth toward the stiffer condition. On the contrary, Piezo1 mediates a low‐frequency influx of Ca^2+^ and then activates NO synthase resulting in vascular extension.^18^ The calpain pathway also contributes to synaptic pruning, which might be regulated by neuronal Piezo1 as well.

### 3.4 TRP channels and ENaC regulate neurovascular coupling

Neurovascular coupling is a crucial mechanism to maintain the supply of metabolic substrates matched well with the energy demand of local neural cells by regulating the local cerebral blood flow via vasoconstriction and vasodilation.^63^ The most well‐researched MSC that regulates neurovascular coupling is TRPV4. Generally, activation of TRPV4 mediates either vasoconstriction or vasodilation, which is complicated and depends on the cell type (i.e., astrocyte, endothelial cells, or smooth muscle cells) and physio‐pathological conditions.^64^, ^65^ The astrocytic endfeet that wrap the parenchymal arteriole express a relatively high level of TRPV4, from where the increased blood flow and/or luminal pressure are translated as TRPV4‐mediated intracellular Ca^2+^ evoke, results in vasoconstriction.^66^, ^67^ The TRPV4‐associated Ca^2+^ events also upregulate cyclooxygenase‐1 and prostaglandin to prevent excessive vasoconstriction.^68^, ^69^ Simultaneously, the astrocytic Ca^2+^ event also activates BKCa channels and triggers an outflux of K^+^, which then activates the Kir channel of smooth muscle cells and causes vasodilation.^65^ The activated astrocytes also release ATP and/or adenosine to increase interneuron firing and decrease pyramidal neuron activity.^69^ Besides, in capillary endothelial cells of the mammalian brain cortex and retina, Piezo1 is over‐expressed and activated when intravascular pressure is increased, and acts as regulators independently without the presence of other purported mechano‐sensors such as TRPV4.^12^

There are many MSCs expressed in the smooth muscle cells of the middle cerebral artery and regulate vasoconstriction caused by high cerebral blood pressure. including TRPC6, TRPM4, βENaC, and γENaC.^26^, ^70^, ^71^, ^72^ Interestingly, all of them show a very similar function in regulating cerebral artery tone. Generally, the high cerebral blood pressure activates those MSCs and introduces Na^+^ and Ca^2+^ influx, followed by opening the voltage‐dependent Ca^2+^ channels, and the high‐level intracellular Ca^2+^ subsequently contracts the actin and results in myogenic vasoconstriction.^73^

On a long time scale, either a gradual reduction or raising of blood pressure contributes to progressive neurodegeneration and dementia.^74^, ^75^ Hypertension activates the MSCs aberrantly, which participate in the neurovascular decoupling‐associated cognitive impairment. Due to the enhanced cerebral blood pressure and/or flow, an upregulated level of TRPV4 is detected in the astrocytic endfeet, which mediates a significantly increased spontaneous Ca^2+^ event in the microdomain of astrocytes. The augmented astrocytic Ca^2+^ events subsequently cause an enhanced parenchymal arteriole tone and vasoconstriction.^76^ Chronic high astrocytic Ca^2+^ are potential risk factors, reasonably because of the excessive excitatory and chaotic astrocytic signals. The phenomenon is reminiscent of the roles MSCs play in blood pressure‐associated neurodegeneration and dementia, possibly through the abnormal activating of MSCs and disturbing the neurovascular coupling, and then causing the starving of neural cells. In conclusion, the MSCs would be promising therapeutic targets for neurovascular uncoupling and diseases caused by abnormal cerebral blood pressure.

### 3.5 Unanswered questions for MSCs in neurophysiological processes

At present, the studies on MSCs in cerebral physiological processes dependent on applying modulators or genetic knockdown systems.^77^ Given that MSCs like Piezo1 also participate in many complex processes that is usually transient and could not be interrogated through current strategies, such as learning and memory, in which the real‐time recording of MSC‐dependent neuronal activities is necessary.^78^ Using strategies such as genetically encoded fluorescent reporter of piezo1,^79^ as well as the in vivo microscopic or mesoscopic imaging, it might be helpful to study the complicated MSC functions in more depth. Developing novel fluorescent reporters for optical recording MSC activity would be also helpful.

Another unanswered question is the potential interaction and the detailed functional differences among numbers of Piezo, TRP, and ENaC families. For example, Piezo1 and Piezo2 play opposite roles in myelin formation, suggesting Piezo1 and Piezo2 might inhibit each other. In addition, TRPC6, TRPV4, TRPM4, βENaC, and γENaC are all observed in responding to cerebral blood flow, but the detailed differences among them are not clear yet.

## 4 THE MSCs RESPOND TO STIFFNESS CHANGES IN NEURODEGENERATIVE DISEASES

### 4.1 The misfolded protein fibrils are much stiffer than local parenchyma while the brain parenchyma turns softer in AD, PD, and multiple sclerosis

The aberrant accumulation of proteins is the feature and biomarker of neurodegenerative diseases. For example, the pathology of Alzheimer's disease (AD) is accompanied by Amyloid‐β (Aβ) plaques and Tau fibrils, and Parkinson's disease (PD) features with the accumulation of α‐synuclein (α‐syn) and the formation of Lewy's bodies.^80^, ^81^ The pathological prion aggregation changes the mechanical properties of AD and PD brains and abnormally activates the MSCs in various cell types. In neurodegenerative patients and mouse models, the accumulated fibrils are significantly stiffer than the brain tissue (Table 2).

**TABLE 2:** The stiffness of the accumulated toxic protein fibril, and the brain with AD, PD, and MS.

Sample resource	Resources	Young's modulus	References
Aβ	Aβ_1–42_ oligomers	1.2 ± 0.5 GPa	^82^
	Aβ_1–42_ protofibrils	2.1 ± 0.6 GPa	^82^
	Aβ_1–42_ fibrils	3.3 ± 0.8 GPa	^82^
	Aβ_1–42_ fibrils	3.2 ± 0.8 GPa	^83^
Tau	Tau fibrils	3.4 ± 0.7 GPa	^83^
	6 types of Tau fibrils isoforms	15.6–34.8 MPa	^84^
α‐syn	α‐syn oligomers	1.5 ± 0.5 GPa	^85^
	α‐syn fibrils	2.2 ± 0.6 GPa	^85^
	α‐syn fibrils	2.2 ± 0.6 GPa	^83^
Health human	Whole scope, 18–35 years	3.5–3.8 kPa	^86^
	Whole scope, 18–33 years	3.23 ± 0.21 kPa	^42^
	Whole scope, 65–72 years	2.5–3 kPa	^86^
	Hippocampus, 18–33 years	3.35 ± 0.30 kPa	^42^
	Hippocampus, 19–30 years	2.89 ± 0.32 kPa	^87^
	Hippocampus, 66–73 years	2.65 ± 0.39 kPa	^87^
	Thalamus, 18–33 years	3.96 ± 0.24 kPa	^42^
	Thalamus, 19–30 years	3.35 ± 0.24 kPa	^87^
	Thalamus, 66–73 years	2.76 ± 0.31 kPa	^87^
AD patient	Probable AD patients	2.2 kPa	^88^
	AD‐like, significant Aβ plaque accumulation	2.32 kPa	^88^
	AD‐like, no Aβ plaque accumulation	2.37 kPa	^88^
	Non‐AD health control	3.07 kPa	^88^
	Cerebrum, 70–87 years, AD	2.23 ± 0.15 kPa	^89^
	Cerebrum, 66–73 years, health control	2.52 ± 0.13 kPa	^89^
	White matter, 70–87 years, AD	2.34 ± 0.19 kPa	^89^
	White matter, 66–73 years, health control	2.65 ± 0.14 kPa	^89^
	Cerebral cortex, 70–87 years, AD	2.02 ± 0.12 kPa	^89^
	Cerebral cortex, 66–73 years, health control	2.33 ± 0.13 kPa	^89^
	Subcortical gray matter, 70–87 years, AD	2.55 ± 0.19 kPa	^89^
	Subcortical gray matter, 66–73 years, health control	2.73 ± 0.23 kPa	43, 89
PD patient	Whole scan, 49–78 years, PD	0.96 ± 0.065 kPa	^90^
	Whole scan, 60–76 years, health control	1.04 ± 0.08 kPa	^90^
	Frontal, 49–78 years, PD	0.99 ± 0.169 kPa	^90^
	Frontal, 60–76 years, health control	1.15 ± 0.214 kPa	^90^
	Striatum, 49–78 years, PD	1.19 ± 0.244 kPa	^90^
	Striatum, 60–76 years, health control	1.24 ± 0.325 kPa	^90^
	Mesencephalic, 49–78 years, PD	0.92 ± 0.116 kPa	^90^
	Lentiform nucleus, 52–74 years, PD	1.96 ± 0.22 kPa	^91^
	Lentiform nucleus, 52–74 years, health control	2.10 ± 0.20 kPa	^91^
MS patient	21–53 years, MS relapsing remitting	3.025 ± 0.459 kPa	^92^
	18–59 years, health control	3.545 ± 0.556 kPa	^92^
	52 (9.1)/51 (5.0) years, MS secondary progressive and primary progressive, respectively	2.607 ± 0.482 kPa	^93^
	48 (9.7) years, health control	3.278 ± 0.068 kPa	^93^
	Whole brain, 22–47 years, clinically isolated syndrome for MS	3.390 ± 0.541 kPa	^94^
	Whole brain, 18–53 years, health control	3.685 ± 0.503 kPa	94, 95

*Note*: Here we list the stiffness (represented as Young's modules) of the Aβ plaques, tau tangles, α‐syn. We also list the stiffness of the brains of the AD patients, PD patients, and the health volunteers to provide a direct comparison between the brain tissues and the pathologically misfolded protein. We also list the decrease of brain tissue stiffness with aging. Here we only summarize the stiffness tested in brain tissue or molecules, mostly by AFM or MRE, but not conclude the simulations and estimations using mathematical and physical models. Here we do not discriminate some physical terms to represent stiffness or elasticity, including Young's modulus, elastic modulus and shear modulus, given the individual difference and inaccuracy of stiffness measurement generally lie in the same order of magnitude of using another modulus and quantified methodologies. The stiffness of a specific brain region is summarized only when the region is related with AD or PD directly.

The Young's modulus of human brain parenchyma lies within the kPa (1 kPa = 10^3^ Pa) ranges; while the Aβ plaques, tau fibrils, and α‐syn are generally more than 10^5^ times stiffer than the surrounding brain parenchyma.^27^, ^83^, ^96^, ^97^, ^98^, ^99^, ^100^ The tau turns longer and stiffer when phosphorylated, and the hyperphosphorylated tau proteins pair inside neurons and constitute neurofibrillary tangles.^84^, ^101^, ^102^ When the α‐syn oligomers transform into mature amyloid fibrils, the random coil is decreased and more β‐sheet content is formed.^85^ The fibrillization of α‐syn with an increment of rigid β‐sheet permits the α‐syn fibrils to reach a very high stiffness, with Young's modulus lying between 1.3 and 4 GPa.^82^, ^103^, ^104^

Though the fibrils provide more intracellular and niche stiffness, the brain with accumulated misfolded protein deposition is not turned stiffer. Instead, Aβ plaques and α‐syn fibrils turn out to aggravate the normal aging‐associated brain softening (Table 2).^43^, ^91^ The mechanism of Aβ plaques‐ and α‐syn‐associated brain softening may differ from the mechanisms that result in cerebral atrophy and degeneration of AD and PD patients, for the obvious softening and degeneration sometimes occur in distinctive brain regions.^89^, ^90^, ^105^ Besides, it is still not clear whether and how and how much the mechanical properties of misfolded fibril proteins participate in accelerating brain softening.

The cerebral softening is also observed in patients with multiple sclerosis, another neurodegenerative disease featuring progressively demyelinating.^106^ The stiffness of the brain is a potential biomarker for reporting multiple sclerosis.^95^

### 4.2 Piezo1, NMDAR, AMPAR, and mitoBKCa respond to the stiff Aβ

The much stiffer fibril deposits in the cytoplasm and extracellular matrix, compress and deform the membrane system, and the compression would also disturb the cytoskeleton. As a result, the MSCs following either the force‐from‐lipids model or the force‐from‐filament model are potentially hyperactivated by the stiff fibril deposits.

**FIGURE 2:**
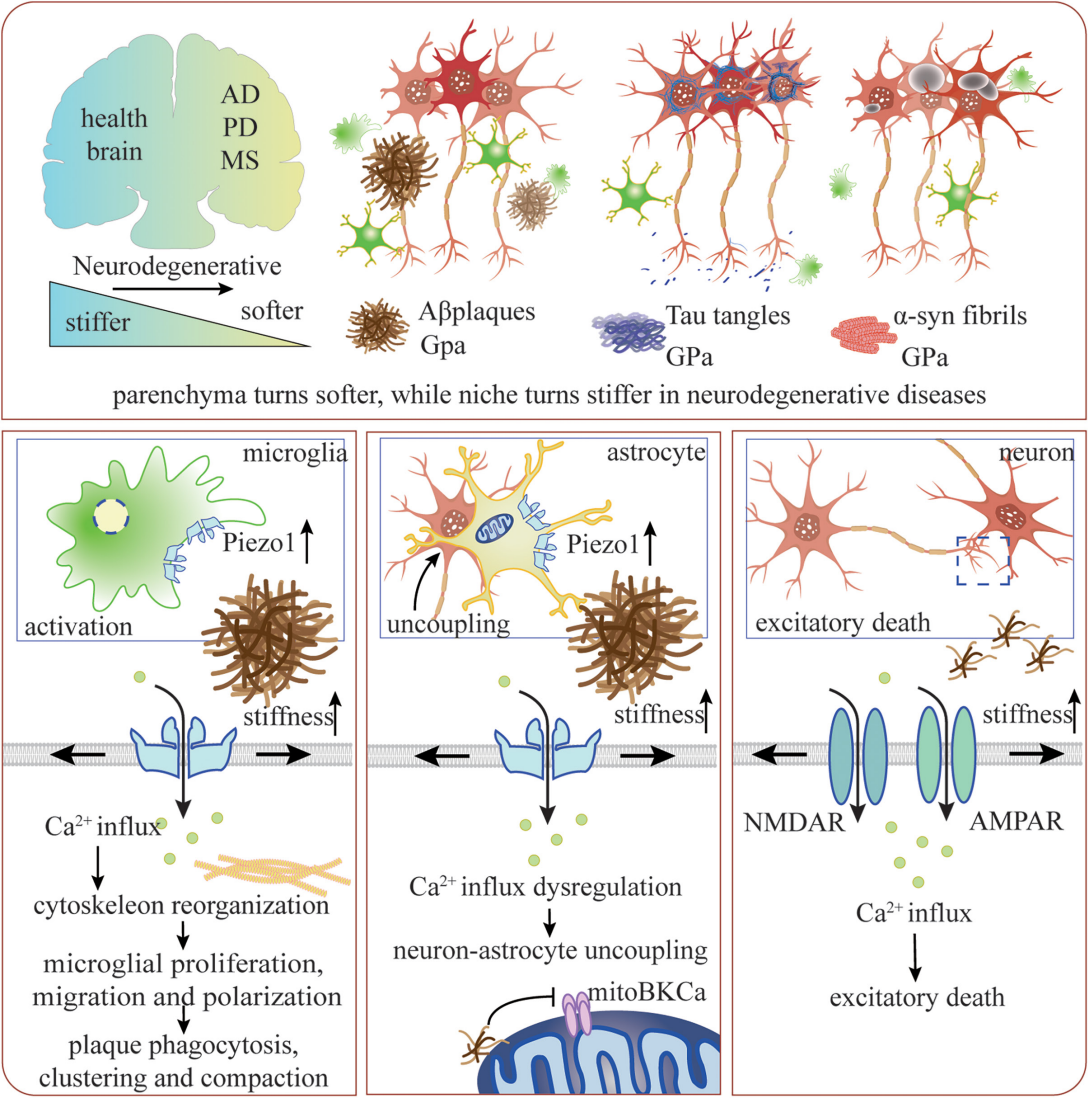
The MSCs respond to the changed stiffness in neurodegenerative diseases. In neurodegenerative diseases including AD, PD, and MS, the brain tissue turns softer, but the niche is stiffened due to the accumulation of misfolded proteins including Aβ plaques, Tau tangles, and α‐syn fibrils (upper). The stiff Aβ plaques alter the MSCs and trigger different downstream pathways in microglia (bottom left), astrocytes (bottom middle), and neurons (bottom right).

The MSCs respond to the Aβ‐related mechanical property changes (Figure 2). Piezo1 expressed in astrocytes and microglia is upregulated in the presence of Aβ plaques.^107^ Primary astrocyte culturing shows that the astrocytes surrounded by Aβ plaques express three times Piezo1 compared with those far away from Aβ plaques, indicating that astrocytes sense the mechanical cues caused by accumulation of Aβ plaques via a Piezo1‐associated mechanism.^108^ Similarly, the upregulation of Piezo1 in microglia localizes mostly in the core of Aβ plaques, and is predominantly triggered by the mechanical but not chemical properties of insoluble Aβ plaques.^96^, ^109^ The upregulation and activation of microglial Piezo1 serve as a protective process against Aβ pathology, because the Piezo1‐activated microglia expresses a high level of genes associated with innate immune response and cytoskeleton dynamics, which introduce the phagocytosis of Aβ and microglial migration, and subsequently result in alleviating Aβ accumulation and cognitive impairment.^96^, ^109^ When downregulating microglial Piezo1, the phagocytic activity toward Aβ is decreased and microglial migration is reduced. Moreover, microglial Piezo1 deficiency decreases the clustering of Aβ plaques and plaque compaction, which might facilitate the migration of Aβ plaques.^96^, ^109^

The NMDAR is one of the most important ion channels in the progressive process of AD,^110^ and participates in NMDAR‐dependent synaptic depression and spine elimination.^111^ For a long time, prevailing studies attribute the phenomenon predominantly to the ligand‐gated properties of NMDAR and the following downstream molecular signaling pathways triggered by different subunits of NMDAR.^112^ However, the mechanical properties of NMDAR and their roles in AD have been ignored.^113^ A similar situation also occurred at AMPAR. When Aβ is deposited on the surface of the membrane, the membrane is stretched, resulting in the opening of NMDAR and AMPAR.^34^ Multidisciplinary studies provide evidence supporting that it is mechanical cues, instead of amyloid fibril or oligomer binding with receptors that gate the channels. Firstly, according to an interactome test, neither NMDAR nor AMPAR are observed to interact with type A or B oligomers of HypF‐N, a convenient protein used to closely mimic the amyloid fibril.^114^ Secondly, the FRET shows that though the Aβ42 oligomers deposit near the NMDAR and AMPAR, they do not interact directly with each other, indicating that the oligomers cause a mechanical stimulation on the membrane and result in opening the NMDAR and AMPAR.^34^, ^115^ The excessive activation of NMDAR and AMPAR would then participate in synaptic dysfunction via complicated downstream pathways.^116^

Besides, the stiff Aβ plaques could disturb the organelle membrane and regulate the MSCs expressed in organelles. The mechanosensitive mitochondrial calcium‐activated potassium (mitoBKCa) channel on the surface of mitochondria was able to be inhibited by all aggregated forms of Aβ.^117^ When Aβ is inserted in the membrane, the membrane phospholipids are packed and the local membrane fluidity is decreased, which is regarded to squeeze on mitoBKCa and close the channels.^117^

In conclusion, our to‐data knowledge on AD‐associated activation of MSCs is mainly focused on the accumulation of the stiff Aβ plaques and the following membrane disturbance. In this way, the neural cells sense the nearby Aβ plaque depositions without interacting with them directly. However, different mechanisms might exist in processes by which Aβ plaques or oligomers activating different MSCs, since the activated threshold of MSCs as well as the stiffness of fibrils would be different. In addition, whether the “non‐binding activation” mechanism could be extended to the other MSCs like Piezo and TRP channels, has not been clarified.

Intriguingly, though the Aβ plaques‐ or fibrils‐associated mechanobiological processes have been interrogated, no studies yet reported whether tau or α‐syn that display similar mechanical properties to the Aβ plaques, affect the MSCs and neuropathological processes, and if so, whether the mechanobiological mechanisms are similar with those triggered by Aβ plaques. Besides, since the Aβ plaques upregulate Piezo1 in several neural cells, whether similar things occur to the other MSCs and Piezo1 expressed in the cerebral vessel epithelium? The potential abnormal Piezo1 and other MSCs in the neurovascular unit would be a risk factor for the damage of neurovascular coupling in neurodegenerative diseases, and the abnormal cerebral blood flow might attenuate the clearance of Aβ, tau, or α‐syn as well.

Besides, since the mechanobiological effects of the stiffer deposits have been reported, the discussion on how the softened parenchyma affects the MSCs in neurodegenerative diseases has not been paid much attention. Multiple sclerosis, for example, is accompanied by loss of parenchyma stiffness, and the downregulation of Piezo1 in oligodendrocytes has been observed, which might be responsible for demyelination in multiple sclerosis.^19^

### 4.3 Changes in parenchyma stiffness occur in other brain pathological processes

Intriguingly, the parenchyma stiffness is also changed in many other brain pathological processes, including brain tumor and epilepsy. Even the infection of SARS‐CoV‐2 would soften cerebral white mass.^118^

The stiffness of brain tumor, however, is different from patients due to the high heterogeneity.^119^ The brain tumors of human patients, including meningiomas, gliomas, vestibular schwannomas, and pituitary adenomas, etc., are generally stiffer than health brain parenchyma.^120^, ^121^ However, there are also gliomas softer than health control.^119^, ^122^ The detailed reasons for the mechanical heterogeneity of brain tumor is still unclarified yet. Since some microRNA is dysregulated in brain tumor,^123^ we suggest that it would be worthwhile to using high‐sensitive strategies in clinical studies to interrogate the correlation between stiffness of brain tumor and microRNA in more detail.^124^, ^125^, ^126^

The mesial temporal lobe epilepsy is associated with changes in hippocampus stiffness. The ratio of stiffness of ipsilateral and contralateral hippocampus of epilepsy patients are significantly higher than that of the healthy controls, indicating a probably dysregulation of MSCs.^127^ Certainly, the mechanosensitive K2P channels participate in epilepsy, though few studies have interrogated their roles mediated by mechanotransduction in epilepsy.^128^ The cortical TREK‐1 level in patients with meningioma‐associated epilepsy was lower than that in meningioma patients without seizures, while the cortical TRAAK level in patients without seizures after meningioma resection was higher than that in patients still with seizures after operation, indicating a potential association among an aberrant mechanical condition given the tumor, the level changes of MSCs, and the seizures, which is deserved to explore in future.^129^

## 5 THE MSCS RESPOND TO FORCES FROM PLACE‐OCCUPYING DAMAGE

### 5.1 The mechanical cues and pathogenesis of place‐occupying damage

**FIGURE 3:**
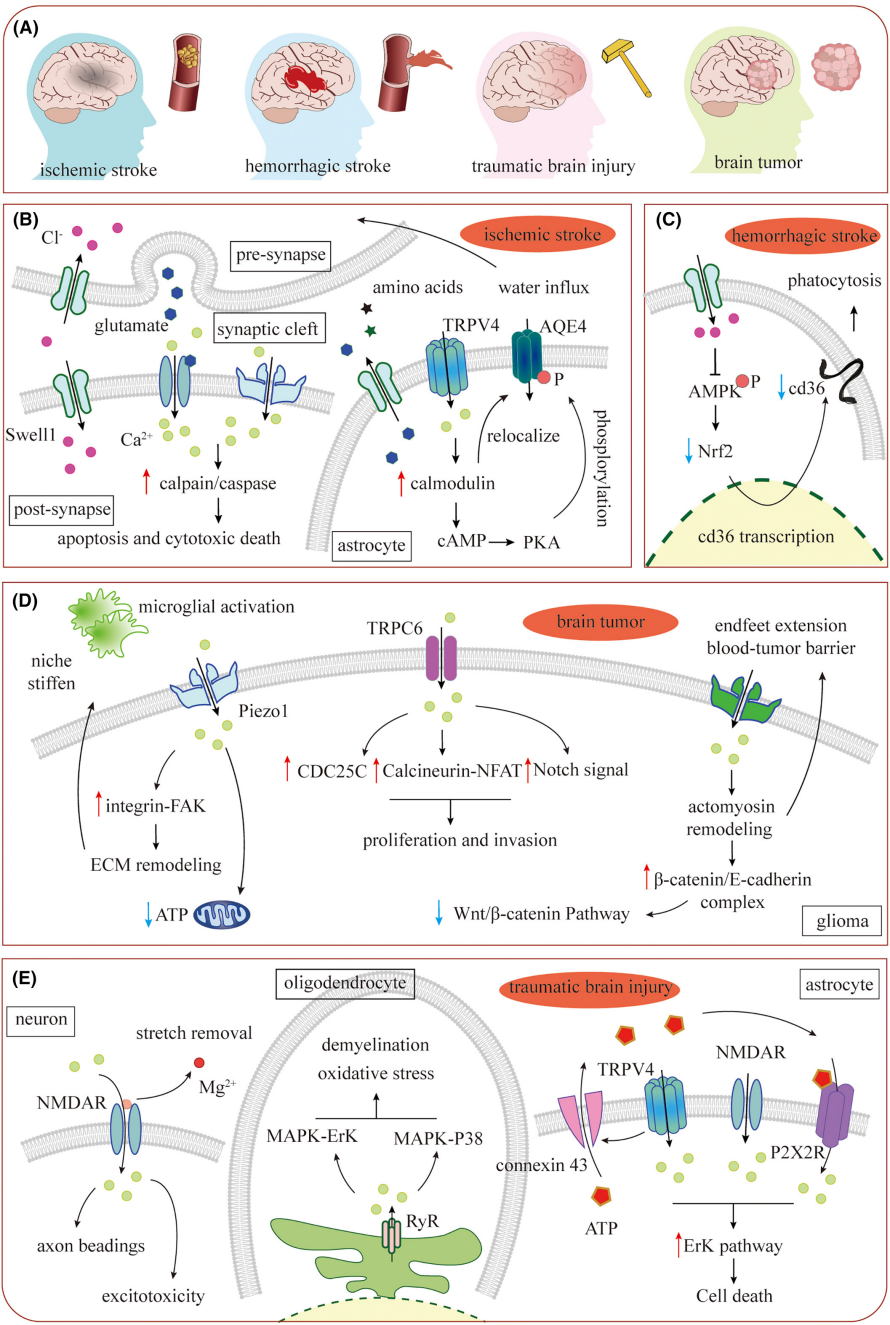
The common mechanisms mediated by MSCs in place‐occupying damage. (A) The brain diseases that cause place‐occupying damages. (B–D) The intracellular signal pathways triggered by the activated MSCs in ischemic stroke (B), hemorrhagic stroke (C), brain tumor (D), and traumatic brain injury (E).

The cerebral place‐occupying damage refers to the lesions expanding into larger chunks that squeeze and deliver a compression onto the adjacent tissue and cause a secondary lesion. Many brain diseases cause place‐occupying damage, including ischemic stroke, hemorrhagic stroke, traumatic brain injury (TBI), and brain tumors, whose place‐occupied bulks are diversified as edema, hematoma, and tumor tissues. The increased compression on the adjacent cells and the pathological activation of the MSCs in various cerebral place‐occupying damage are generally common, though the downstream pathways triggered by MSCs in different cells are distinct (Figure 3).

The place‐occupying damage is usually accompanied by cerebral cytotoxic edema. The cytotoxic edema is feathered with water influx mediated by glial and neuronal Aquaporin 4 (AQP4), whose volume‐sensitive properties are thought to be regulated by the other MSC. The membrane stretching caused by cellular swelling activates TRPV4 and leads to an influx of Ca^2+^, followed by activating calmodulin, a Ca^2+^‐binding protein with sophisticated functions. The activated calmodulin interacts with adenylyl cyclase and activates protein kinase A, results in phosphorylating AQP4 and transporting them onto the membrane.^130^ The increment of activated AQP4 results in the exacerbation of cytotoxic edema. The deletion of TRPV4 decreases the cell volume in oxygen–glucose deprivation, while knockout of both TRPV4 and AQP4 even restrains the cell swelling.^131^ The activation of TRPV4 also contributes to the release of glutamate, which would cause excitotoxicity.^132^

### 5.2 Swell1 and Piezo1 trigger pathological processes in stroke‐caused edema

Cerebral ischemic stroke is caused by cerebrovascular blocking. Ischemia results in oxygen and glucose deficiency, which then deactivates the ATP‐dependent ion pumps such as Na^+^‐K^+^ ATPase, causing a net influx of Na^+^, Cl^−^, and H_2_O, and finally leads to ionic cellular edema. The physical barriers of CNS (i.e., blood–brain barrier) would be disrupted during stroke and reperfusion, leading to plasma and cerebrospinal fluid contents leaking into the cerebral parenchyma, and finally inducing vasogenic edema.^133^ The hemorrhagic stroke causes cerebral bleeding and the concretionary blood acts as the place‐occupied bulk.

The MSCs respond to these mechanical squeezing. The swelling after ischemic stroke activates neuronal and astrocytic Swell1. In neurons, the excitotoxic Cl^−^ influx at both pre‐ and post‐synapses is accompanied by glutamate releasing and activation of apoptosis‐related pathways, respectively.^32^ In astrocytes, glutamate was released through Swell1 directly.^14^ The increased extracellular glutamate then exacerbates the adjacent neuronal excitotoxic cell death and contributes to the brain injury.^14^ However, recent research argued that there would be other Swell1‐associated mechanisms that are responsible for neuron death after stroke. Because the protection is yielded only in the whole brain Swell1 deletion mouse but not in the astrocytic Swell1 conditional knockout mouse.^13^ Blocking Swell1 decreases the release of glutamate, aspartate, and taurine significantly in an in vivo model of hypo‐osmotic‐mediated swelling. In the experimental ischemic stroke model, however, only the decreased release of taurine is observed.^13^ In hemorrhagic stroke, the microglial Swell1 is upregulated. The activation of microglial Swell1 decreases the phosphorylation of AMP‐activated protein kinase activation and finally inhibits the phagocytosis of microglia and exacerbates the neural damage.^33^ However, microglial Swell1 does not affect prognosis and microglia morphology in ischemic stroke.^134^

Piezo1 is also associated with ischemic stroke and affects the pathological processes via various mechanisms.^135^ In patients with risk factors of ischemic stroke like hypertension and hyperglycemia, Piezo1 is upregulated and activated in several blood cells, including platelets, red blood cells, neutrophils, and hematopoietic stem cells.^136^, ^137^ Hypertension and mechanical squeezing activate platelet Piezo1 and accelerate platelet clotting by upregulating PI3K/AKT and phosphatidylserine.^136^, ^137^ Moreover, the enhancement of shear forces occurs at the narrowed vessel with thrombosis, which might further activate Piezo1 constantly and deteriorate the embolism. The upregulation and activation of Piezo1 would last after ischemia and reperfusion in neurons, and participate in apoptosis by triggering Ca^2+^/calpain signaling.^138^ In the following neuropathological processes such as place‐occupying edema, Piezo1, and other MSCs might also be activated perpetually due to the tissue squeeze, and in this hypothetic situation, neuronal excitotoxicity death or ferroptosis would be aggravated.^139^

### 5.3 Piezo and TRP channels respond to the compression of brain tumors result in proliferation, escaping, and inflammation

The brain tumor tissues with an elevated stiffness could invade into the adjacent tissues including neural cells and blood vessels, and apply high compressive stress.^28^ Emerging evidence supports that various MSCs localized at brain tumors or the tumor‐caused edema are upregulated, including TRPC6, Piezo1, and Piezo2. Piezo1 have been suggested as potential prognostic biomarkers for the early diagnosis of glioma,^140^, ^141^ given the level of Piezo1 is positively correlated with the severity of edema and the degree of compression forces.^142^, ^143^, ^144^

The activation of TRPC6 enhances the intracellular Ca^2+^ signaling and then facilitates the cell cycle progression to promote cell proliferation by activating the Notch pathway.^24^, ^25^ The elevated Piezo1 localizing at focal adhesions of the glioma processes enhances Ca^2+^ influx, which promotes glioma invasion by triggering the integrin‐FAK signaling.^145^ In a common mechanical condition of brain tumor, the oxidation of fatty acid in the glioma cells produces ATP, but since a severe squeeze presents or a sonodynamic therapy is given, more Ca^2+^ influx is introduced, resulting in an enhanced level of mitochondria reactive oxygen species which peroxides the fatty acids and decreases the ATP production.^52^ Simultaneously, the tremendous activation of Piezo1 decreases the extracellular Ca^2+^, which introduces the macrophage M1 polarization and causes cerebral inflammation.^52^

The glioma tissue also occupies the place of blood vessels. The cerebral vessels are squeezed and would be turned narrow, so that the local blood perfusion would be reduced. The compression on the vessel wall promotes wrapping the local capillaries to plunder nutrition, and the construction of a blood‐tumor barrier promotes escaping from the immune system and drug.^20^ the activated Piezo2 triggers endothelial WNT/β‐catenin pathway but not those in medulloblastoma Sox2+ cells, which wraps the local capillaries to form the blood‐tumor barriers. In the endfeet of the tumor cells, the influx of Ca^2+^ enhances the extension of mechanosensitive endfeet via promoting actomyosin remodeling and adhesion at the growth cones.^20^

### 5.4 NMDAR and TRPV4 mediate excitotoxicity caused by extreme mechanical force in TBI

TBI is caused by an extreme external mechanical force applied on the brain and serves as the leading cause of disability, dementia, and mortality.^146^, ^147^, ^148^, ^149^, ^150^ The damage in cerebral parenchyma is primarily induced by mechanical forces, which, subsequently, cause a series of pathological processes such as axonal injury, synaptic loss, demyelination, degeneration, excitotoxicity, and cell death.^151^

The TBI‐caused stretch and shear forces break and disassemble the axonal cytoskeleton.^152^, ^153^, ^154^ The stretch on the membrane helps remove the Mg^2+^ that blocks the channel of NMDAR and thus activates them.^5^, ^155^, ^156^ The NMDAR‐mediated Ca^2+^ influx participates in the formation of the beadings after traumatic axonal injury, but the detailed mechanisms are not clear.^157^ Besides, the excessive activation of NMDAR contributes to excitotoxicity.^5^

The tremendous mechanical stretch causes oligodendrocyte degeneration and demyelination. The exogenous forces deformed the myelin directly.^158^ In cultured oligodendrocytes, mechanical stretch induces a spike of intracellular Ca^2+^ within 10 s, predominately by opening the ryanodine receptor, an ER Ca^2+^ channel with mechanosensitive properties in pathological conditions.^36^, ^37^ The high level of intracellular Ca^2+^ activates MAPK‐ErK1/2 and MAPK‐p38 signaling pathways, finally resulting in oxidative stress‐caused cell death and demyelination.^159^, ^160^ Reported in other cell lines, the MAPK pathways could also be activated by stretch‐induced opening of Piezo1 and TRPV4, and these MSCs might also participate in demyelination after TBI via similar mechanisms.^161^, ^162^

In astrocytes, there is another stretch‐activated ErK pathway after TBI. The astrocytic TRPV4 mediates an influx of Ca^2+^, and an unclarified interaction between TRPV4 and connexin 43 hemichannels (the subunits of gap junction), results in ATP releasing from connexin 43 channel.^163^ The extracellular ATP, which accompanies the astrocytic mechanic sensor caveolae‐associated protein caveolin‐1, activates P2 purinergic receptors, and aggravate Ca2+ influx.^164^, ^165^, ^166^ The transients of intracellular Ca^2+^ subsequently trigger ErK pathways and lead to cell death.^164^, ^165^, ^166^

Intriguingly, to our knowledge, it seems that neural and glial Piezo1 or Piezo2 are absent in transducing the mechanical signal in TBI. Maneshi et al. reported that the Ca^2+^ influx could not be affected by GsMTx4, but could be inhibited by Gd^3+^, indicating that the stretch‐activated mechanosensitive Ca^2+^ channels are not members of the Piezo family.^166^

Besides, mild mechanical stimulation would be beneficial to health. Exercise (i.e., running) has been reported to regulate the cortex mechanically, is there any MSC that could be activated by daily exercise, and whether the activation beneficial to health?^167^

## 6 MANIPULATING MSCS WOULD BE A NOVEL THERAPEUTIC METHODOLOGY FOR BRAIN DISEASES

### 6.1 Reasons for targeting MSCs in brain diseases

The MSCs would be promising therapeutic targets for treatment. The MSCs are upstream triggers for the complicated and difficult‐to‐manipulated intracellular biochemical processes in neural diseases, making them potential targets for effectively regulating several signaling pathways associated with the diseases. Given a specific MSC participates in several neuropathological processes, as well as the roles the MSC plays in these processes are generally common, a specific MSC‐targeted drug might present multiple implementations for different neural diseases.

In the past decades, potential strategies to manipulate MSCs in the brain have been developed. There has been an ever‐increasing list of accessible regulators for MSCs. The regulators include neuropeptides and small molecules, providing diversified choices for clinical application in different conditions. For example, GsMTx4, a natural neuropeptide, is an allosteric inhibitor for cationic MSCs like Piezo1, TRPC1, and stretch‐activated BKCa.^168^, ^169^ In contrast, Yoda1, a small molecule, is an agonist for Piezo1.^170^ Besides, many regulators for TRP channels have been developed, which was concluded in a detailed review.^171^

### 6.2 Mechanisms for using LIUS to manipulate MSCs

Beside using biochemical molecules as regulators for MSCs, physical stimulation strategy, LIUS, is another method to manipulate the activation of MSCs, which has been already used in clinical trials. LIUS has advantages in non‐invasiveness, high spatial resolution, depth penetration, and no direct induction of electric field or current in the brain, compared with other neuromodulation methods used in clinical treatment.^172^ Currently, the specific mechanisms of ultrasound neuromodulation have only been interrogated in animal models or cultured cells. The MSCs reported to be regulated in vivo or in vitro by this strategy include Piezo1, TRPA1, TRPC1, ENaC, ASIC1a, and TRAAK.^173^, ^174^, ^175^, ^176^, ^177^, ^178^, ^179^, ^180^ There is also some other research using non‐neural cell lines to confirm the feasibility of ultrasound‐caused modulation of MSCs.^6^

In recent years, studies focusing on the cellular and molecular mechanisms of ultrasound modulating neural activity has increased rapidly, but it has also brought new discussions and controversies. It has been confirmed that the neuromodulation effect of ultrasound on the CNS is mainly based on regulating MSCs. Briefly, LIUS delivers mechanical forces through the intact skull into the deep brain regions, thus activating or inhibiting the MSCs.^6^, ^181^ For example, the ultrasound‐induced opening of TRAAK share similar submillisecond kinetics compared with canonical mechanical activation, indicating that the ultrasound transduce energy and deform the membrane thus activate TRAAK,^175^ given the opening of TRAAK follows force‐from‐lipid model.^31^ However, the molecular processes or kinetics of ultrasound modulation of MSCs following force‐from‐filament model such as Piezo1, have not been clarified.

The ultrasound‐induced activation of MSCs then amplitudes the neural signals. For example, in awaking mouse, the ultrasound activates astrocytic TRPA1, and the increased astrocytic Ca^2+^ then opens astrocytic Best1 channel to release glutamate into the synaptic cleft, resulting in activating NMDAR and eventually regulating neuronal activity.^166^ The influx of Ca^2+^ in neuron Ca^2+^ could be amplified by other channels like NMDARs to produce a burst firing.^178^ However, another study using mesoscopic cortical Ca^2+^ functional imaging in mouse under an ultrasound stimulation showed that there might not be a direct Ca^2+^ response induced by ultrasound, indicating that there would be other potential mechanisms in ultrasound neuromodulation.^182^

It should be emphasized that various MSCs would be regulated by ultrasound simultaneously, so that difficulty is left on evaluating the effect of a specific MSC produces in the overall outcomes. Genetic deletion experiments showed that Piezo1 plays irreplaceable roles in ultrasound‐induced neural activity and motor behavior.^162^, ^183^ However, another research shows that in cultured neuron, it is knockdown of TRPC1, TRPP1, and TRPP2, instead of Piezo1, significantly weakened the neuron activity under ultrasound stimulation.^178^ The different mediators participated in ultrasound neuromodulation would be attributed to the different relative expression level of the MSCs in different cerebral regions or cell types, the parameters (i.e., frequency and intensity) of the ultrasound, and the differences in activity thresholds of those MSCs, and even the natural firing state or patterns of the neurons.^184^, ^185^ It is reported that the ultrasound with same parameters can either inhibit neuronal firing at low spontaneous firing frequency or low input current in CA1 pyramidal neuron of mouse brain slice, or potentiate firing at high spontaneous firing frequency or high input current, probably through the K2P channels.^185^

Recently, the ultrasound stimulation, for the first time, is confirmed able to regulate a specific neurological and physiological response by activating a specific MSC in a specific brain region. Briefly, Yang et al.^186^ using remote transcranial ultrasound to stimulate neurons of hypothalamus preoptic area precisely to induce the torpor‐like state, while downregulating TRPM2 channel suppresses these responses.

### 6.3 The clinical trials using LIUS treating neural diseases

Though emergingly increased research provide evidence that LIUS contributes to neuromodulation via activating MSCs, and LIUS has been attempted in treating brain disease of human patients, the clinical trials using LIUS, to our knowledge, seem never been aimed and designed to regulate a specific type or family of MSCs yet.^187^ The gaps between the basic and clinical studies attribute to the lack of methodologies for interrogating the idiographic activation of MSCs in human brains, and the lack of knowledge of detailed processes and mechanisms trigger by LIUS.

Here we discuss some representative studies using LIUS in human subjects. In healthy people, utilizing 500 kHz transcranial focused ultrasound to stimulate the unilateral inferior frontal gyrus for 30 s or 2 min increase self‐reported emotional states.^188^ The transcranial ultrasound stimulation targeting unilateral prefrontal cortex promotes an approach‐like risky behavior.^189^ The potential mechanism in mesoscopic level might lie on the downregulation of the resting‐state network connectivity associated with emotion,^188^ and the decrease of midfrontal theta activity that is associated with several psychiatric diseases.^189^, ^190^ When using a daily repeated ultrasound with the same frequency and duration but a lower ultrasound power on depressed participants, trait worry level, the chronic feeling of nervousness, is decreased, while the other quantitative indexes for evaluating depression are not changed.^191^

Besides, LIUS has been used in treating epilepsy. In a recent pilot clinical trial, patients with mesial temporal lobe epilepsy are treated with transcranial ultrasound targeted the hippocampus. It is reported that the seizure frequency is decreased after ultrasound treatment for a long period, from weeks to more than one year, and no significant side effects are reported.^192^

### 6.4 The potential side effects for manipulating MSCs and promising implementations

As discussed above, the feasible methodologies for manipulating MSCs in the brain including utilizing LIUS and applying specific inhibitors, antagonists or agonists. Ultrasound neuromodulation has been proven to be safe in human subjects since no significant side effects were reported, suggesting that manipulating a variety of MSCs simultaneously accompanied by some other processes, would be safe. Nevertheless, whether the clinical effects as well as the reported side effects of LIUS are attributed to regulating MSCs, instead of altering other processes, are not clarified yet.

However, applying the modulators for MSCs might introduce serious adverse events. The precise and effective delivery across the blood–brain barrier toward the target regions using a non‐invade strategy is still difficult but necessary. This is because the wide expression and multifunctional roles of the MSCs in both nervous systems and the other organs make it unfeasible to inject modulators of MSCs systemically or oral administration, for the side effects would be caused by affecting MSCs in the other organs like liver and kidney.^171^ Some clinical trials using antagonists of TRPV1 systemically caused a fever. As a result, novel targeting drug carrier should be developed.^193^, ^194^

The existing MSC modulators are not very specific (i.e., GsMTx4 inhibit Piezo1, Piezo2, BKCa, and TRPC1, etc.), providing probability to inhibit a wide number of hyperactivated MSCs, as well as leaving problems of miss‐targeting. For example, given Piezo1 participate in neurophysiological processes and recognition discussed above, the miss‐targeting and abuse of GsMTx4 would also dysregulate these crucial processes. The different expression level between brain regions and individual difference among patients would also cause an uncertain intervention outcome.

In case of this condition, since GsMTx4 is a peptide, it would be worthwhile to express it directly in specific neurons of the specific brain region where MSCs are hyperactivated pathologically, through cerebral stereotactic injecting recombinational adeno‐associated virus or lentivirus coded GsMTx4, or even transplanting neural stem cell expressing GsMTx4. This is because GsMTx4 selectively inhibits Piezo and TRP channel families and given that the changes in parenchyma stiffness or compression would hyperactivate several MSCs simultaneously, the continuous synthesis of endogenous GsMTx4 would provide a prolonged inhibition of the hyperactivated MSCs in chronic brain diseases. The potential low membrane permeability might be improved by fusing with another gene‐encoded peptide titled “TAT”, a cell penetrating peptide composed of 10 amino acids, which have been used in peptide drug development for brain diseases.^195^

Evidence have shown that the expression of MSCs in various brain regions are different, which would impact the targeted treatments of brain diseases.^196^ Generally, the expression level of MSCs depends on the local mechanical conditions and specific regions or nucleus. For example, Piezo2 expression presents significant positive correlations with stiffness in human white matter.^196^ In the regions with place‐occupy damage or fibril deposition, the level of MSCs is usually upregulated as discussed above. The focal high level of MSCs would enhance the therapeutic effectiveness of LIUS. The future studies should further interrogate the spatiotemporal distribution and activation of MSCs in developing and aging brains in detail, as well as the correlation between patterns of mechanical condition and MSCs, to provide more necessary information to instruct drug developing and clinical treatment.

## 7 CONCLUSION

In summary, MSCs widely contribute to health and diseases in the brain. The common mechanisms mediated by mechanical cues and MSCs provide the MSCs with promising therapeutic targets for multiple neurological disorders. This current review sheds light on the fundamental and translational research of the MSCs in the future.

## AUTHOR CONTRIBUTIONS

The manuscript was conceived by Bolong Li and Xin Yang, and written by Bolong Li and Xin Yang with contributions from An‐ran Zhao and Tian Tian.

## CONFLICT OF INTEREST STATEMENT

There are no conflicts of interest.

## Data Availability

Research data are not shared.
